# Bridging barriers: a mixed-methods study on using health communication to increase knowledge on mental health services among migrant parents in Sweden

**DOI:** 10.1136/bmjph-2025-003272

**Published:** 2026-08-05

**Authors:** Ana Hagström, Anna-Clara Hollander, Karima Viksten Assel, Henna Hasson, Hanna Augustsson Öfverström

**Affiliations:** 1 Learning, Informatics, Ethics and Management (Lime), Karolinska Institutet, Stockholm, Sweden; 2 Centre for Epidemiology and Community Medicine, Stockholm Health Care Services, Region Stockholm, Stockholm, Sweden; 3 Department of Global Public Health, Karolinska Institutet, Stockholm, Sweden

**Keywords:** Community-Based Participatory Research, Mental Health, Program Evaluation, Social Medicine, Qualitative Research

## Abstract

**Abstract:**

**Background:**

Children with a migrant background are at increased risk for mental health issues. Despite this, they often use mental healthcare less than the majority population in the host countries. This study evaluated if participating in a multilingual health communication for migrant parents improved their self-reported knowledge of children’s primary mental healthcare, influenced their perceived barriers to accessing care and if it was perceived as relevant and acceptable.

**Methods:**

The health communication was implemented in a diverse municipality in Stockholm, Sweden. We applied a convergent parallel mixed-methods design to collect data with questionnaires before the intervention, immediately after and at 4-month follow-up, together with interviews with a subsample of parents at follow-up. Main outcome variables were knowledge on where to seek mental healthcare, perceived barriers to mental healthcare and acceptance and relevance of the health communication.

**Results:**

A total of 101 parents participated in the health communication, with 80 completing the baseline questionnaire and 47 completing the follow-up. Parental knowledge of where to seek mental healthcare for their children increased significantly after the intervention and participants reported less shame associated with seeking care and fewer language-related difficulties at follow-up. In the interviews, the parents described having limited knowledge of mental health in children and available mental health services. They appreciated the health communication, including learning about available mental health services for children and found it well aligned with their needs.

**Conclusions:**

This study showed that health communication may reduce individual-level barriers, such as limited knowledge of available services and low mental health literacy, while also improving parental awareness and perceptions of seeking care for their children. The findings suggest responsiveness and motivation among parents for similar interventions.

**WHAT IS ALREADY KNOWN ON THIS TOPIC**

- Children with a migrant background in high-income countries experience a higher risk of mental health problems but use mental health services less often than children in the majority population. 
- Barriers such as limited knowledge of the health system, language difficulties and stigma may hinder access to mental healthcare for migrant families.  

**WHAT THIS STUDY ADDS**

- Migrant parents reported low mental health literacy and limited knowledge of where to seek mental healthcare for their children prior to the intervention. 
- The study shows that health communication can target modifiable individual-level barriers to mental healthcare access for migrant children, particularly parents’ limited knowledge of the healthcare system. 
- This study provides insights from an underserved group of parents not already engaged with healthcare services. It suggests that challenges in navigating the healthcare system, rather than stigma, may be a more important barrier to accessing mental healthcare, and indicates positive attitudes towards health communication.

**HOW THIS STUDY MIGHT AFFECT RESEARCH, PRACTICE OR POLICY**

- Multilingual health communication interventions may be a feasible public health strategy to reduce individual-level barriers and promote equitable access to child mental health services among migrant families. 
- The study suggests that interventions should prioritise increasing knowledge among both parents and healthcare staff, rather than focusing primarily on stigma-related barriers.
- Scaling up and integrating such interventions within local health and community settings could strengthen early engagement with mental healthcare and support preventive efforts.

## Introduction

 Both refugee children and non-refugee migrant children have an increased risk of adverse mental health outcomes. Refugee children particularly have higher risks of depression, anxiety and post-traumatic stress disorder,^1,2^ whereas non-refugee migrant children face mental health risks related to the psychosocial stress of migration, acculturation, discrimination and low socioeconomic living situations.^3–5^ If left untreated, mental illness in children is associated with poor health including mental health, difficulties in school and with social interactions frequently continued into adulthood.^6^ Despite their heightened risk of mental illness, children with a migrant background underuse outpatient specialist mental healthcare and are less frequently diagnosed with psychiatric conditions as compared with their peers without migrant background.^7–11^ At the same time, use of emergency psychiatric services and inpatient care is higher among refugee and migrant children than among majority peers.^8,10,12^

The reasons for these disparities are not entirely clear but barriers to accessing appropriate levels of healthcare are likely to play an important role.^13,14^ Migrant-specific challenges such as difficulties navigating the healthcare system, limited awareness of available services, language barriers and inadequate provision of accurate information by services are known contributors to unequal access.^13,15^ In addition, mental health can be a sensitive topic in some cultural contexts, and concerns about stigma may discourage some migrants from seeking care.^15^

Inequalities in utilisation may be reduced by addressing these barriers.^1,13,16^ In relation to children’s mental health, increasing parental awareness of children’s mental health and available services is an important factor to target.^17–19^ Evidence-based interventions aiming to increase healthcare-seeking behaviour among migrants in host countries remain limited in both scope and quality.^1,20^ However, a scoping review found that health communication is a common approach to improving care-seeking among migrants and found that such interventions are most effective when tailored to the needs and context of specific migrant groups.^20^

### Health communication to promote health among migrant populations

Health communication refers to group-based sessions for individuals who share similar background characteristics, such as language, and are designed to inform and engage participants on health-related issues, with the goal of promoting health at both individual and societal levels.^21^ This approach may be particularly important for migrant families, who often face language barriers, limited knowledge of the health system, stigma around mental health and mistrust of authorities. Health communication has the potential to raise awareness of available services, build trust in health services, improve access and reduce stigma by improving understanding of mental health problems and normalising help-seeking. It may also enhance families’ experiences of healthcare encounters.^14,20,22^ Moreover, culturally tailored parent-support and health communication interventions have been associated with improved health literacy, stronger parental practices and better family well-being.^23^

However, whether health communication can increase parental awareness of mental health services for children with a migrant background or help overcome barriers to seeking and using mental healthcare remains underexplored. This study therefore aimed to assess whether participating in a multilingual health communication intervention for migrant parents improved self-reported knowledge of children’s primary mental healthcare, influenced perceived barriers to accessing care and was perceived by participants as relevant and acceptable.

## Materials and methods

This study is part of a multicomponent community programme called *the ‘bridging barriers to care for children with a migrant background’* (TINA with acronym in Swedish) see study protocol.^24^ For this study, a child with a migrant background was defined as an individual aged 0–18 years whose two parents were forcibly displaced migrants with residence permits granted on protection grounds and who had lived in Sweden for no more than 10 years.

### Study design

A convergent parallel mixed-methods design was used to compare perceived healthcare barriers before and after participation, using longitudinal questionnaires at three time points. Interviews complemented the questionnaires to explore perceived barriers and the relevance and acceptability of the health communication. The Template for Intervention Description and Replication Checklist^25^ was used for describing the intervention and COREQ for reporting the qualitative data (online supplemental file 1).^26^

### Setting

The health communication took place in a demographically diverse municipality in the Stockholm region, with nearly 100 000 residents—28.7% with a migrant background in 2021, compared with 20.0% nationally.^27^ Around 14.2% lived in socioeconomically challenged neighbourhoods, compared with 9.8% in Stockholm.^28^ The municipality has received asylum seekers for over 30 years and hosts one of the largest primary mental healthcare services for children. In Sweden, child mental healthcare is organised at two levels: mild to moderate conditions are treated in primary healthcare, while more severe illness and neuropsychiatric assessments are referred to specialist services.

### Developing the health communication

The health communication was adapted from the health communication programme for immigrants, arranged by the regional knowledge centre for migration and health.^29^ It has been delivered over the past decade by bilingual ‘health communicators’ educated in nursing/medicine/equivalent. The same health communicators delivered the health communication evaluated in this study.

The adapted version of health communication aimed to raise awareness of children’s mental health and available support, with the longer-term goal of promoting care-seeking (online supplemental file 2). The content was developed by an experienced child and adolescent psychiatrist and an experienced child psychologist. The work was supported by an advisory group including an experienced health communicator and the manager of the municipality’s primary mental healthcare centre. A reference group of experts in migrant child mental health reviewed the content for consistency with national guidelines across age groups. The health communicators provided feedback on format and delivery to ensure relevance for newly arrived parents. The final version included five themes: (1) normal development vs signs of mental distress in children and adolescents; (2) barriers to care from a migrant perspective; (3) the structure of Swedish mental health services; (4) the role of the psychologist; and (5) how to contact primary mental health services.

### Piloting the health communication

The health communicators participated in five digital trainings before the pilot, led by the child psychiatrist and psychologist. In addition to the content, the training addressed the role of the health communicator and confidentiality. A coalition was formed with the municipal integration unit from the social services, the family-health central, the centre for Swedish for Immigrants (SFI) and TINA researchers to identify suitable venues for piloting. The content, recruitment strategies, delivery format (digital/non-digital), venues and evaluation questionnaires were piloted in nine sessions with 49 participants, including 20 who tested the questionnaires, in Arabic, Somali, Tigrinya and Dari.

The original five themes of the health communication were retained after piloting, the original two separate modules—one for younger and one for older children—were merged into one, as it was difficult to cover both within the session time and many parents had children of different ages. The evaluation questionnaires were found too long by both participants and staff and were therefore shortened and retested. The revised version was used for the final evaluation.

Digital and hybrid formats for providing the health communication proved unsuitable for discussion, as some parents lacked email accounts, had limited internet access or felt uncomfortable with digital interaction. Venues included ‘SFI’ classes, the municipal’s parental support groups for migrants (‘Föräldrarskap i Sverige’ (FÖS)), toddler–parent groups and faith communities. Recruitment was challenging and FÖS proved the most suitable setting as newly arrived parents were already attending regular sessions held at the family health central. FÖS is a civic orientation programme for foreign-born parents, comprising five 2.5-hour sessions with themes on family life in Sweden, children’s rights, gender equality and role of school and Swedish welfare and health system.^30^

### Public and patient involvement

We involved health communication facilitators in developing the material. The staff from the municipal integration unit were involved from the outset in planning the health communication, disseminating information and recruiting eligible parents. A representative from the local community, a multilingual integration coach, was also involved after pilot testing the health communication, hereafter called the coordinator. The coordinator played an important role in recruitment, information dissemination and communication about the intervention. They supported data collection by distributing and collecting study materials, providing study and consent information, sharing contact details and collecting participants’ contact information for follow-up. They were available for practical support when parents responded to the evaluation questionnaire but did not influence participants’ responses or intervene in completion of the questionnaire.

Parents or other public representatives were not involved in assessing the burden associated with participation, including intervention activities and time commitment, nor were they involved in disseminating the study results. Aggregated findings from this study, together with findings from the wider TINA intervention, were shared with the public at a community and policy event, including a panel discussion on inequitable access to mental healthcare. Representatives of the target population were invited to participate as audience participants.

### Participants

Eligible parents were those who had lived in Sweden for no more than ten years and held a residence permit based on protection needs. Participation in health communication was voluntary. Recruitment was carried out with support from the municipal integration unit responsible for FÖS and the coordinator, who informed parents about the purpose of the health communication during municipal parent-support gatherings. Information was also posted on the municipality’s website. Parents who wished to participate signed up with the FÖS facilitators or the coordinator, who managed the registration. SMS reminders were sent to parents who had consented to receive them. For sessions held in local libraries, the coordinator again informed parents during parent-support gatherings. Typically, one parent per family attended. All parents who participated in the health communication were informed about the evaluation study and invited to take part. Informed consent was obtained before participation.

### Implementing the health communication

The health communication sessions were delivered in 2022–2023 to 12 separate face-to-face groups, with each session delivered as a one-time event lasting approximately 2.5 hours (online supplemental file 3). Each group included 6*–*12 parents. The health communication was delivered either as the final session of FÖS or as standalone sessions arranged by the coordinator at the local library.

Sessions were held in Arabic, Dari/Farsi, Somali and Tigrinya. To support implementation, a psychologist provided counselling to the health communicators during the first semester of the roll-out in spring 2022, as the topic was new to many of them. The content was delivered using PowerPoint and presented through a combination of lecture, case-based examples, dialogue and video.

### Data collection

Data were collected using questionnaires and individual interviews. Respondents answered questionnaires at three time points: just before attending the health communication (T0), directly after (T1)—both at the venue for the health communication. Follow-up questionnaires and interviews approximately 4 months after the health communication (T2) were conducted over the telephone. Participants who consented to participate in the 4 month follow-up questionnaire and interview provided their contact details. Follow-up questionnaires were conducted as a structured interview by telephone, the only feasible method, by bilingual research assistants who administered both the questionnaire and interviews. In total, 47 participants completed the follow-up questionnaire and 27 of them participated in an interview. Interview informants were selected based on expressed interest; those who declined mostly did so due to lack of time or interest. The interview sample reflected the broader participant demographics (table 1).

**Table 1:** Participant characteristics

Category	Description	N (%)			
		Baseline, T0 (N=80)	Postintervention, T1 (N=79)	Follow-up, T2 (N=47)	Interviews (N=27)
Sex	Male	31 (38.8)	30 (38.0)	23 (48.9)	12
	Female	49 (61.3)	49 (62.0)	24 (51.0)	15
Years in Sweden	1–5 years	48 (60.0)	47 (59.5)	29 (61.7)	15
	6–10 years	31 (38.8)	31 (39.2)	17 (36.2)	11
	Missing	1 (1.25)	1 (1.26)	1 (2.12)	1
Participated in FÖS	Yes	42 (52.5)	42 (53.2)	29 (61.7)	19
	No	35 (43.8)	34 (43.0)	15 (32.0)	6
	Missing	3 (3.8)	3 (3.8)	3 (6.4)	2
Education	No formal education or incomplete primary education	44 (55.0)	43 (54.4)	25 (53.2)	11
	Elementary completed	19 (23.8)	19 (24.0)	10 (21.3)	6
	High school completed	10 (12.5)	10 (12.7)	9 (19.1)	6
	University degree	7 (8.8)	7 (8.9)	3 (6.4)	2
Language	Arabic	21 (26.3)	21 (26.6)	14 (29.8)	4
	Dari/Farsi	32 (40.0)	31 (39.2)	13 (27.6)	7
	Tigrinya	11 (13.8)	11 (13.9)	8 (17.0)	7
	Somali	11 (13.8)	11 (13.9)	8 (17.0)	9
	Other	5 (6.2)	5 (6.3)	4 (8.5)	0

### Variables and outcome measures

#### Participant characteristics

Information on participants’ demographic characteristics was collected at baseline and included sex, education, spoken language and time of stay in Sweden (years).

#### Barriers to mental healthcare and knowledge of mental health services

Barriers to mental healthcare were assessed with questionnaires at baseline (T0) and follow-up (T2) and included barriers related to language, navigating the health care system and mental health stigma. Barrier items on cost and long waiting times to healthcare were assessed only at baseline to determine whether these were general perceived barriers, as they could not be influenced by the health communication. Knowledge of where to seek mental healthcare was assessed at all three time points.

As there were no validated questionnaires for assessing barriers to mental healthcare, single-item measures were developed based on barriers identified in literature on real and perceived barriers faced by migrants in host countries.^14,31^ An example question was: “I would find it difficult to seek help for mental health issues within healthcare because I feel it is shameful to suffer from mental illness.” The response alternative was a Likert scale from completely disagree (1) to completely agree (5).

#### Acceptance and relevance of the health communication

Acceptance of and perceived relevance of the health communication was assessed directly after the health communication (T1) using a previously developed questionnaire.^32^ The response alternative was a Likert scale from completely disagree (1) to completely agree (5).

All questionnaire items were translated following guidelines for cross-cultural translation and included back-translation, peer-check and pilot testing with participants.^33^

### Interviews

Audio-recorded semistructured interviews (30–40 min) were conducted with a subset of participants via telephone to explore barriers, relevance and perceptions of health communication (interview guide online supplemental file 5). The interviews were conducted in the informants’ native languages by research assistants. All interviewers were female. AH, who had briefly met some of the informants during the intervention, trained the research assistants on interview techniques, ethics and coordinated the interviews. The research assistants were not involved in any other aspects of the implementation and evaluation of the health communication and had no prior relationship with the participants.

### Data analysis

#### Statistical methods

We used descriptive statistics for demographic characteristics and to calculate means and medians for the pre and post questionnaires. Non-parametric Wilcoxon signed-rank tests and the Friedman tests were used to compare differences over time. Some variables had missing responses, which were included as missing data in the statistical analyses. To examine potential differences between participants who completed the follow-up and those who dropped out, we conducted Pearson’s χ^2^ tests across all demographic characteristics and outcome variables. Dropout analysis was also performed to identify correlations between participants’ acceptance data and their continuation in the study. R (V.4.2.2) was used for analysis.

#### Qualitative interview data

The research assistants transcribed and translated the interviews. Content analysis with an inductive approach^34^ was used. All transcripts were read several times, and meaning units related to the study aim and research questions were identified and coded by AH. Coded segments were sorted into subcategories and categories by AH and HAÖ, then compared against the transcripts to ensure alignment with the original data. This iterative process involved checking whether the data, emerging categories and synthesised text accurately reflected the raw material.

## Results

### Participant characteristics

Of the 101 parents who participated in the health communication, 80 (78%) completed the baseline and 79 (78%) completed the postintervention acceptance and perceived relevance of the health communication. A total of 47 participants (47%) completed the follow-up (table 1).

Mean years in Sweden was 4.8 years. Most participants were mothers, and education levels ranged from none to university, with the majority having no formal or incomplete primary education. Most had also participated in the FÖS parental support programme. Eight languages were represented, with bilingual participants using one of the four health communication languages—Arabic, Dari, Somali or Tigrinya.

Dropout analysis identified two statistically significant differences: male participants and those who had participated in FÖS were more likely to remain in the study (p=0.046 and p=0.037, respectively) (details in online supplemental file 4). Attrition was not associated with acceptance reported immediately after the health communication.

### Perception of barriers to healthcare

At baseline, participants generally rated Swedish healthcare more positively than healthcare in their country of origin (median: 5.0 vs 4.0), (table 2). While most parents knew where to seek care for physical illness in their child (median: 4.0), fewer reported knowing where to seek help for mental health problems (median: 2.0). Perceived barriers such as stigma, language difficulties and negative treatment by health staff were rated low overall.

**Table 2:** Baseline questionnaire

Survey question	Mean	Median (IQR)	N
I think the healthcare in my home country is good	3.48	4 (3.0)	77
I think the healthcare in Sweden is good	4.70	5 (0.0)	77
If my child is physically ill I know where to seek healthcare	4.03	4 (1.0)	77
If my child has mental problems (sleeping problems, aggressions, depression), I know where to seek healthcare	3.03	2 (4.0)	79
I think it is easy to book an appointment at the health centre	3.27	4 (3.0)	76
I have sought healthcare when my child or I needed it	3.40	4 (2.0)	75
I worry that sensitive information about me or my child will be shared with people other than healthcare staff	1.77	1 (1.0)	75
I would rather solve my health problems in other ways than seeking healthcare	1.60	1 (0.25)	76
I would find it difficult to seek help for mental health issues within healthcare because I feel it is shameful to suffer from mental illness	2.07	1 (2.0)	76
I have avoided seeking healthcare because I think it is difficult to speak Swedish	2.50	2 (3.0)	78
I have avoided seeking healthcare because I have been treated badly by healthcare staff	1.34	1 (0.0)	78
When I have needed healthcare, I have avoided seeking it due to long waiting times	2.77	3 (3.0)	77
I have avoided seeking healthcare because I needed my money for other expenses	1.74	1 (1.0)	78

### Comparison baseline and follow-up perceived barriers to healthcare

We found statistically significant decreases in the perception that healthcare in Sweden is good (p=0.0038), and in feelings of shame associated with seeking healthcare for mental distress (p=0.0001), and in the tendency to avoid healthcare due to a language barrier (p=0.0437). A statistically significant increase was observed in whether participants reported seeking healthcare for themselves or their child when needed (p=0.0098). No significant differences were observed in the other items on barriers (table 3).

**Table 3:** Comparison of parents’ perceived barriers to healthcare

Survey question	Baseline n=47		Follow-up n=47		P value
	Mean	Median (IQR)	Mean	Median (IQR)	
I think the healthcare in Sweden is good	4.75	5.0 (0.0)	4.19	5.0 (1.0)	0.0038*
I think it is easy to book an appointment at the health centre	3.22	4.0 (3.0)	3.78	5.0 (3.0)	0.0676
I have sought healthcare if myself or my child needed healthcare	3.95	4.5 (2.0)	4.60	5.0 (0.0)	0.0098*
I worry that sensitive information about myself or my child will spread to others than healthcare staff	1.73	1.0 (0.75)	1.57	1.0 (0.0)	0.5999
I would rather solve my health problems in other ways than seeking healthcare	1.66	1.0 (1.0)	1.29	1.0 (0.0)	0.1488
I would find it difficult to seek help for mental health issues within healthcare because I feel it is shameful to suffer from mental illness	2.22	1.0 (2.0)	1.36	1.0 (0.0)	0.0001*
I have avoided seeking healthcare because I think it is difficult to speak Swedish	2.46	2.0 (3.0)	1.82	1.0 (1.75)	0.0437*
I have avoided seeking healthcare because I have been treated badly by healthcare staff	1.37	1.0 (0.0)	1.19	1.0 (0.0)	0.5502

* Significance level was <0.05. T0=baseline, T1=directly after intervention, T2=after 3–5 months. Scale responses were: 1=completely disagree to 5=completely agree.

The item “I know where to seek healthcare if my child has mental health problems (sleeping problems, aggressions, depression)” was measured at all three time points (table 4) and showed a statistically significant increase in knowledge about where to seek mental healthcare for their child (p=0.013).

**Table 4:** Comparison of parents’ perceptions on knowing where to seek mental healthcare for their children

Survey question	Time	Mean	Median	IQR
If my child has mental health problems (eg, sleep issues, aggression, depression), I know where to seek healthcare	T0	3.22	4.0	3.0
	T1	4.43	4.5	1.0
	T2	3.72	4.0	2.5
	Friedman χ^2^=8.71, df=2, p=0.013*			

* Statistically significant at the 0.05 level. Table 4 is based on complete cases

### Ratings of acceptance and relevance of the health communication

Participants’ ratings of acceptance of and perceived usefulness of the health communication were high across all items with mean values ranging from 4.6 to 4.9 (median 5.0) (table 5).

**Table 5:** Acceptance and relevance of the health communication

	Mean	Median (IQR)	N
I found the level of difficulty in the health communication to be good	4.56	5 (1.0)	78
I feel that the health communication contained information that I need to know	4.64	5 (0.5)	79
I will have practical use for what I have learnt during the health communication	4.75	5 (0.0)	79
I trust the group leader’s competence	4.86	5 (0.0)	79

### Qualitative results

Five categories and fourteen subcategories (online supplemental file 6) were developed to capture perceptions of how relevant the health communication was for receiving information on children’s mental health, the mental healthcare system and explore experiences of Swedish healthcare access. The categories were: expanding knowledge and gaining practical insights; purpose comprehension; enhancing mental health literacy; uncertainty about mental health; and perceptions of healthcare access in Sweden.

#### Expanding knowledge and gaining practical insights

Parents reported gaining important insights, appreciating the opportunity to learn about mental healthcare for their children, and understanding the role of mental health services.

First, I thought it seemed unnecessary, but then I understood it was educational. […] It was good to learn more about mental health and where to turn to because I thought the primary health centre was only for somatic issues, Man, 3 years in Sweden

They valued gaining guidance on how to book appointments, how to communicate with healthcare staff and contact healthcare services in Stockholm, including mental health services for children. This extended to a deeper understanding of mental health problems and realising it is common. Several parents expressed they were not that familiar with mental health, and that it was unheard of earlier.

I had never heard of mental illness before I came to Europe and it was good that I learned more, Man, 3 years in Sweden

However, some participants found it less relevant and believed that it would have been more useful with other themes.

It was a good opportunity to learn something. Although I was hoping they could have brought up other themes. Like CPR or swimming for example, Man, 8 years in Sweden

Parents perceived the *purpose* of the health communication as a help for navigating the Swedish healthcare organisation, gaining insights into children’s mental health but also how to manage as a parent. While the majority appreciated and understood this purpose, some saw it as irrelevant, particularly those with very young children and some believed it was a requirement in the Swedish language introduction course given to immigrants. Others suggested that the health communication might be more applicable to parents of teenagers or to teenagers themselves.

#### Perceptions of and suggestions for improvement of the health communication

The format and content of the health communication were generally found to be well presented and clearly communicated. The combination of dialogic pedagogy with supportive PowerPoints containing text, pictures and videos was appreciated, and many participants felt their questions were adequately addressed. This approach, along with the opportunity to discuss topics with the facilitator and peers, fostered the sharing of experiences and insights, and several suggested offering more than one session.

It was good that we could discuss, and that way help each other, learning from each other was an advantage, Woman, 4 years in Sweden

It was so nice to listen […] talking openly about the information, Man, 4 years in Sweden

Suggestions of alternative formats also came up—such as online lectures or radio broadcasts to expand accessibility. The need for translated materials and videos, to enable viewing calmly at home and at one’s own own pace, or together with their children, came up.

[…] it should be in written so that for those who absorb information slowly, can go over the material in a calm and slower pace at home, Woman, 4 years in Sweden

Conversely, some suggested information only orally, not in writing

I also think this information should be orally rather than in writing since many of us have problems in reading, Woman, 2 years in Sweden

Concerns about the sessions being too fast-paced and brief were noted, with suggestions to modify the group size to either include more participants for broader perspectives or fewer for deeper discussions.

What worked well was that there was an interpreter there and that you could ask questions if something was unclear. What didn't work well was that it went too fast and I didn't really feel that I got answers to the questions I had, Man, 9 years in Sweden

#### Enhancing mental health literacy

Mental health literacy varied among the parents, some encountered similar information to that from their home countries, while others gained new understanding about navigating the mental healthcare system and on parenting and mental health in children, specifically focusing on improving communication and managing distress.

In my home country there is little known about mental health, it seems so well-known in Sweden and now when we live here it is important to learn about this. We should seek Swedish healthcare that understand this much better, rather than us doing “Quran saar”, Woman, 5 years in Sweden

Several stressed the importance of addressing their children’s mental health concerns and consulting experts when necessary, including social services, and the importance of supporting other parents with similar problems and pointing out that seeking help is not shameful.

I would advise my friend to not feel ashamed or stigmatized […] and I would talk to my child […] and if we cannot solve it, contact a psychologist, Woman, 2 years in Sweden

#### Uncertainties on the concept of mental health

Most parents expressed uncertainty about mental health because they had not heard much about it or considered it taboo in their home country. Some parents reflected on how these traditions and views on psychiatry could influence parents’ willingness to seek mental healthcare. Moreover, religion could play a part where mental health symptoms were associated with bad behaviours caused by the devil.

Views on psychiatry and regarding it as a disease that can be cured through religion, seeking priests, the condescending view of the sick person and their family. There is a ’shame stigma' associated with visits to psychiatric care, where some […] eventually struggle on their own and do not seek professional help because revealing mental health issues can be embarrassing due to the social stigmatization that affects the family. Woman, 3 years in Sweden

Scepticism about the motives behind health communication provided by public authorities was also communicated.

Unnecessary with information about mental health, this is just made up by the Swedish state to lock up [take into care] all foreign children, Woman, 5 years in Sweden

On the other hand, several expressed the opposite and would seek help from professionals. Several also emphasised the role of school staff and the importance of collaboration between schools and municipal authorities for the sake of their children’s mental well-being.

[Us parents need to] discuss and get evaluation about our children from school and healthcare, Woman, 2 years in Sweden

#### Health communication improved understanding, but trust in the healthcare system remains an issue

The category *perception of healthcare access in Sweden* comprised experiences of long waiting times and communication issues, which were perceived barriers in healthcare encounters. Interpreters were helpful, though some participants found their role confusing and felt that information could be lost in translation.

There’s an interpreter — I have seen one at the child care or doctors — but no one ever explains what it actually means to use one, Man, 3 years in Sweden

Some had benefited from physicians speaking their native language or support from friends. Despite barriers, many described positive experiences and felt well treated. Navigating the system—especially understanding urgency and when to seek care—was challenging, but most felt the health communication improved their understanding and provided useful guidance. Some noted that care quality had improved over time, while others still expressed low trust in healthcare staff due to earlier negative experiences, affecting their willingness to seek further help.

Ehh… here you are sent home with “Alvedon” and “Ipren” [painkillers] […] feel that the Swedish healthcare system thinks you are weak if you seek care for mild conditions… I have therefore chosen not to seek care when I get sick but of course I go there when the children get sick, Woman, 2 years in Sweden

## Discussion

This study assessed if participating in a multilingual health communication for migrant parents improved their self-reported knowledge of primary mental healthcare for children and influenced their perceived barriers to accessing care. An additional aim was to see if the parents perceived health communication as relevant and acceptable.

### Health communication to increase awareness of mental health and available care services

Many appreciated discussing sensitive topics in their native language in a supportive setting. These findings align with previous research on the benefits of culturally adapted approaches to increase health literacy.^15,35^

Interviews revealed participants’ limited understanding of mental health and lack of awareness of primary mental healthcare. Despite this, most were open to learning more and willing to seek care if needed, and there was an increased knowledge of where to seek care, along with a reported reduction in language-related barriers and shame associated with seeking mental health support. Although the sample size limits definitive conclusions, these findings are promising.

The low awareness aligns with literature showing asylum seekers and refugees often turn to somatic rather than psychiatric care, despite high rates of depression and posttraumatic stress disorder (PTSD).^2,15,36^ This mismatch in health need and reaching adequate service can be attributed both to stigma and taboo related to mental health symptoms but more likely to lacking awareness of mental health services.^17,37,38^ A recent review found that limited health literacy remains a strong barrier and that 60%–70% of the foreign-born population in Europe faces challenges navigating the healthcare system.^38^ Although some expressed low interest, most parents emphasised the importance of raising awareness about the Swedish mental healthcare system. The high acceptance ratings in the post-intervention questionnaire also reflect this perspective. These findings suggest that health communication may be a promising way to increase newly arrived migrants’ knowledge on mental health services.

### Challenges in navigating the healthcare system, rather than stigma, hinder access to mental healthcare

In contrast to several studies reporting on mental health stigma as a barrier to accessing mental healthcare,^6,14,31,39,40^ our study found low baseline questionnaire ratings related to stigma and even lower ratings at follow-up. Also, the interview findings illustrated openness and interest in learning more about mental health services for children. This aligns with a previous study where parents who had sought mental healthcare for their children did not report stigma as a barrier, but rather a lack of knowledge on how to initiate contact. Referral agents, such as school staff, had helped them find the appropriate service and navigate care.^17^ Both that study and this study’s participants acknowledge stigma as a societal problem in their home countries, consistent with findings that migrant parents are more open to seeking mental healthcare in resettlement countries than in their countries of origin.^41^ Olsson *et al*^42^ found that waiting for mental health problems to resolve on their own and negative experiences with Swedish healthcare were more common reasons for not seeking care than stigma. In the present study, parents expressed a desire for help from experts if needed, for stronger collaboration between schools and social services to prevent future mental health problems, despite coming from contexts where such issues are often not addressed in healthcare or are considered taboo. Clear referral pathways between schools and healthcare have been found important for improving access to first-time mental health consultations for immigrant children.^43^ Low awareness of mental health issues and services—as well as difficulties contacting services, language barriers and limited mental health literacy—even when services are known, are reported as barriers rather than stigma-related obstacles.^15,37,44^

### Awareness gaps and structural barriers in migrants’ mental healthcare

Some interviewees reported mistrust and concerns about not being taken seriously by healthcare. Interestingly, while more participants indicated that they had sought care for themselves or their children, the questionnaire rating “I think the healthcare in Sweden is good” declined between baseline and follow-up. A potential explanation may be that before health communication, negative experience in healthcare was perceived as individual (or random). When exchanging experiences with peers, negative experiences may have been seen more as a structural problem with the Swedish healthcare system. This aligns with evidence on limited understanding of migrants’ specific needs among healthcare staff,^16^ along with negative care experiences, contributes to perceptions of unmet healthcare needs among migrants.^45^ Efforts to increase migrants’ knowledge of mental health services should be combined with training to strengthen healthcare staff’s cultural competence.^46,47^ That is important since structural or relational barriers can persist, such as perceived discrimination or culturally mismatched care, that continue to affect migrants’ experiences of healthcare access, particularly after the initial ‘honeymoon phase’ of resettlement has passed.^13,48,49^

Given the group’s diversity in terms of education, country of origin and different experiences with authorities, it is reasonable to assume they faced different barriers. Research links high perceived discrimination to poorer mental health, although this relationship is moderated by a strong sense of belonging.^50^ Individuals who identify with both their home and host countries tend to report better mental health outcomes. Similarly, learning the host country’s language and integrating aspects of its norms—while maintaining one’s ethnic identity—supports psychological well-being.^50,51^ Future health communication efforts should therefore be tailored to address these varied needs, whether by building trust, enhancing mental health literacy or supporting positive identity integration.

### Implementation considerations and lessons learnt

Although the small sample size limits definitive conclusions, these findings are promising. High levels of acceptance among participants indicate a receptiveness to health communication efforts, reinforcing the importance of community-based strategies to improve mental health literacy.

Recruitment was challenging, as the intervention targeted a population that is often underserved and difficult for health systems to reach; involving the coordinator who had lived experience of migration facilitated recruitment. Implementation was resource-intensive, and success hinged on strong local anchoring. Despite a bottom-up approach during the planning phase, including needs assessment interviews and collaboration with the municipal integration team and local mental health services, external factors such as COVID-19 and high staff turnover in the municipality limited broader institutional engagement. As a result, much of the initiative remained anchored in the research team.

These findings highlight the importance of strong local ownership. Given the challenges in recruitment, anchoring the intervention within the parent community, particularly through parents with a migrant background, would likely increase its relevance and improve engagement. Key lessons learnt point to the value of participatory strategies. First, establishing an advisory group of parents with a migrant background could provide guidance on the development of information materials, recruitment strategies and the format and content of health communication. Second, this advisory group, together with the local research assistant, could support the dissemination of findings and updated information on mental health, thereby fostering stronger engagement and more sustained relevance within the community.^52^

### Strengths and limitations

This study has several strengths and limitations other than mentioned above. It is uncommon to find interventions targeting multiple language groups; this study reports on the evaluation of health communication across several languages. Migrants from non-European countries are generally underserved in health research at the group level, and this study enabled their perspectives to be captured regarding a widely used—but rarely evaluated—format: health communication. Given the diverse sample, representing a broader group of parents in the community, not those already engaged in clinical care, and the low levels of stigma reflect positive attitudes. This suggests that interventions should prioritise increasing knowledge among both parents and healthcare staff, rather than focusing primarily on stigma-related barriers.

The small sample size did not allow for group comparisons based on demographic variables in the analysis of baseline and follow-up data. With a larger sample, it would have been possible to examine differences between the Somali-speaking, Eritrean-speaking, Arabic-speaking and Dari-speaking/Farsi-speaking groups, as well as between participants with different levels of education. Such an analysis could offer insights into potential nuances across groups—differences that, while not evident in the limited and relatively similar quantitative responses, were more apparent in the interviews. As participants represented four distinct language groups and social contexts, their diverse backgrounds likely influenced how they experienced the health communication and their perceptions of encountering the Swedish healthcare system. Despite this, the interviews illustrate changes in reported barriers and offer deeper insight into parents’ perceptions of mental healthcare encounters, which is a strength that contributes to this field.

The health communication was delivered as a one-time intervention, and the study design did not permit long-term follow-up. Thus, it is not possible to draw any conclusions about how the health communication impacts actual mental healthcare seeking. This is in line with findings by Al-Adhami *et al*,^23^ who reported that health communication embedded in Sweden’s civic orientation improved health literacy among newly arrived refugees, though broader health outcomes were not affected.

The use of a self-developed questionnaire may limit the generalisability of findings. Self-reported data are also prone to bias, including social desirability and varied interpretation based on educational level.

## Conclusion

This study showed that a multilingual health communication session may reduce individual-level barriers, such as limited knowledge of available services and low mental health literacy. The findings suggest responsiveness and motivation among parents for similar interventions. Future research should examine whether repeated sessions lead to actual healthcare seeking, ideally through longitudinal studies or linkage to healthcare utilisation data.

## Supplementary material

10.1136/bmjph-2025-003272 online supplemental file 1

10.1136/bmjph-2025-003272 online supplemental file 2

10.1136/bmjph-2025-003272 online supplemental file 3

10.1136/bmjph-2025-003272 online supplemental file 4

10.1136/bmjph-2025-003272 online supplemental file 5

10.1136/bmjph-2025-003272 online supplemental file 6

## Data Availability

Data are available upon reasonable request.
