# Supplementary material for: Bridging barriers: a mixed-methods study on using health communication to increase knowledge on mental health services among migrant parents in Sweden
Source: BMJ Public Health. 2026 Aug 5;4(3):e003272. doi: 10.1136/bmjph-2025-003272 (PMC13448519; doi:10.1136/bmjph-2025-003272)
Supplement: Supplementary file 1 [file bmjph-4-3-s002.pdf]

Assumptions/ background

Higher prevalence of mental health disorders in newly arrived children

Under-utilisation of mental health care in newly arrived children

Resources

Health communicators

Collaboration municipal key actors for recruitment

Local health care venue to boost familiarity with services

Standardized PPT

Activity

Health communication about mental health in children 0-18 y. for newly arrived parents

Content

- Normal development compared with signs of mental distress among children and adolescents;
- Potential barriers to care from a migrant perspective
- Swedish mental health care levels and available mental health services
- The role of a psychologist
- How to contact 1st mental health services.

Format

- Group meeting IRL
- Native language
- Dialogue pedagogic
- 2 ½ hours

Outcome Short

Improved knowledge and awareness among parents on

- Mental health literacy in children
- on 1st line mental health services for children

Decreased perception of barriers to mental health care

Output: Satisfaction with health communication & Perceived relevance of health communication

Outcome Intermediate

Increased mental health care seeking at 1st line mental health care services when signs of mental distress

Impact

Improved mental health in target population
