# Supplementary material for: Bridging barriers: a mixed-methods study on using health communication to increase knowledge on mental health services among migrant parents in Sweden
Source: BMJ Public Health. 2026 Aug 5;4(3):e003272. doi: 10.1136/bmjph-2025-003272 (PMC13448519; doi:10.1136/bmjph-2025-003272)
Supplement: Supplementary file 2 [file bmjph-4-3-s001.docx]

# Supplementary File 1. COREQ Checklist: Consolidated Criteria for Reporting Qualitative Research

| **Domain 1: Research team and reflexivity** | | Reported on Pg |
| --- | --- | --- |
| 1. Interviewer/facilitator: Which author/s conducted the interview or focus group? | The interviews were conducted by trained research assistants with support from the first author (AH) | 5 |
| 2. Credentials: What were the researcher's credentials? | RN, PhD candidate (AH), senior researchers (ACH, HAÖ), Professor (HH) | N/A |
| 3. Occupation: What was their occupation at the time of the study? | RN, PhD candidate | 5 |
| 4. Gender: Was the researcher male or female? | The first author is female. | 5 |
| 5. Experience and training: What experience or training did the researcher have? | The first, second and third authors have extensive experience in migrant health. Two last authors are experts in implementation research in public health | N/A |
| 6. Relationship established: Was a relationship established prior to study commencement? |  | 3,4,5 |
| 7. Participant knowledge of the interviewer: What did the participants know about the researcher? | Participants were informed of the researcher’s role and the purpose of the study. | 3,4 |
| 8. Interviewer characteristics: What characteristics were reported about the interviewer/facilitator? | The interviewers were trained in ethical and culturally sensitive qualitative data collection and were matched linguistically | 5 |
| **Domain 2: Study design** | |  |
| 9. Methodological orientation and theory: What methodological orientation was stated to underpin the study? |  | 2,4 |
| 10. Sampling: How were participants selected? | Participants were selected from those who had taken part in the health communication sessions and agreed to participate in a follow-up interview. | 4,5 |
| 11. Method of approach: How were participants approached? | Participants were contacted by telephone for follow-up and invited to participate in the interviews | 4,5 |
| 12. Sample size: How many participants were in the study? | 80 in the total mixed method study, 27 in interview study | 4,6 |
| 13. Non-participation: How many people refused to participate or dropped out? | 33 dropped-out from the original 80 in the baseline questionnaire. 47 participated in the follow-up questionnaire, 27 of those participated in an interview | 4,6 |
| 14. Setting of data collection: Where was the data collected? | Interviews were conducted by telephone. | 5 |
| 15. Presence of non-participants: Was anyone else present besides the participants and researchers? | Since data was collected over phone in the informants home, family members to the informant were home in some cases | N/A |
| 16. Description of sample: What are the important characteristics of the sample? | Participants were migrant parents with children, representing diverse languages and backgrounds. See Table 1 for demographics pg 7. | 2, 4, 6,7 |
| 17. Interview guide: Were questions, prompts, guides provided by the authors? Was it pilot tested? | Yes, a semi-structured interview guide was used with prompts. It was pilot tested. Supplementary file 5 | 5 |
| 18. Repeat interviews: Were repeat interviews carried out? | No repeat interviews were conducted. | N/A |
| 19. Audio/visual recording: Did the research use audio or visual recording to collect the data? | Audio-recording | 5 |
| 20. Field notes: Were field notes made during and/or after the interview or focus group? | No | N/A |
| 21. Duration: What was the duration of the interviews or focus group? | Interviews lasted approximately 30–40 minutes. | 5 |
| 22. Data saturation: Was data saturation discussed? | Yes, although since there were four different interviewers no comparison was possible during data collection, researcher checked data for saturation | N/A |
| 23. Transcripts returned: Were transcripts returned to participants for comment and/or correction? | No, transcripts were not returned to participants. | N/A |
| Domain 3: Analysis and findings | | |
| 24. Number of data coders: How many data coders coded the data? | Two authors were involved in coding the data. | 5 |
| 25. Description of the coding tree: Did authors provide a description of the coding tree? | Yes, the coding tree is included as Supplementary File 6 | 9 |
| 26. Derivation of themes: Were themes identified in advance or derived from the data? | Categories were drawn inductively from the data. | 5,9 |
| 27. Software: What software, if applicable, was used to manage the data? | Excel and manually conduction | N/A |
| 28. Participant checking: Did participants provide feedback on the findings? | No, participant checking was not carried out. | N/A |
| 29. Quotations presented: Were participant quotations presented to illustrate the themes/findings? | Yes, representative quotations are included to support findings. | 9-12 |
| 30. Data and findings consistent: Was there consistency between the data presented and the findings? | Yes, the findings are grounded in the data and supported by illustrative quotes. | 9-12 |
| 31. Clarity of major themes: Were major themes clearly presented in the findings? | Yes categories and subcategories. | 9 |
| 32. Clarity of minor themes: Is there a description of diverse cases or discussion of minor themes? |  | N/A |
|  |  |  |
