# Supplementary material for: Bridging barriers: a mixed-methods study on using health communication to increase knowledge on mental health services among migrant parents in Sweden
Source: BMJ Public Health. 2026 Aug 5;4(3):e003272. doi: 10.1136/bmjph-2025-003272 (PMC13448519; doi:10.1136/bmjph-2025-003272)
Supplement: Supplementary file 3 [file bmjph-4-3-s003.docx]

**Supplementary file 3.** Overview health communication sessions

|  | Year | N of planned groups | N implemented | Of those that took place | |
| --- | --- | --- | --- | --- | --- |
|  |  |  |  | Within FÖS | Standalone |
| Pilot | 2021 | 11 | 9 | 5 | 4 |
| Intervention and evaluated sessions | **2022** | 11 | 7 | 1 | 6 |
|  | **2023** | 5 | 5 | 3 | 2 |
