# Supplementary material for: Bridging barriers: a mixed-methods study on using health communication to increase knowledge on mental health services among migrant parents in Sweden
Source: BMJ Public Health. 2026 Aug 5;4(3):e003272. doi: 10.1136/bmjph-2025-003272 (PMC13448519; doi:10.1136/bmjph-2025-003272)
Supplement: Supplementary file 4 [file bmjph-4-3-s004.docx]

# Supplementary File 4. Table dropout Between Baseline (T0) and Follow-up (T2)

| Variable | Dropped out¹ (n) | Completed follow-up (n) | p-value |
| --- | --- | --- | --- |
| Gender |  |  | 0.0456* |
| Male | 8 | 25 |  |
| Female | 23 | 24 |  |
| Participation in FöS |  |  | 0.0374* |
| Yes | 13 | 29 |  |
| No | 20 | 15 |  |
| Education |  |  | 0.1632 |
| None/Started elementary | 19 | 25 |  |
| Elementary completed | 9 | 10 |  |
| High school completed | 1 | 9 |  |
| University degree | 4 | 3 |  |
| Language |  |  | 0.1544 |
| Arabic | 7 | 14 |  |
| Dari/Farsi | 19 | 13 |  |
| Tigrinya | 3 | 8 |  |
| Somali | 3 | 8 |  |
| Other² | 1 | 4 |  |
| Years in Sweden |  |  | 0.7970 |
| 1–5 years | 19 | 29 |  |
| 6–10 years | 14 | 17 |  |

¹ "Dropped out" refers to participants who completed the baseline survey (T0) but not the follow-up (T2).

² "Other" includes Dinka, Kurdish, and Pashtu speakers; these participants were multilingual and participated using one of the HC languages.

* Statistically significant at the 0.05 level.

Pearson’s chi-squared tests were used for all comparisons.
