# Supplementary material for: Bridging barriers: a mixed-methods study on using health communication to increase knowledge on mental health services among migrant parents in Sweden
Source: BMJ Public Health. 2026 Aug 5;4(3):e003272. doi: 10.1136/bmjph-2025-003272 (PMC13448519; doi:10.1136/bmjph-2025-003272)
Supplement: Supplementary file 5 [file bmjph-4-3-s005.docx]

**Supplementary File 5**

Interview guide short semi-structured telephone interviews after participating in health communication

1. How did you experience the health communication session?
 *Interviewer prompt: What worked well? Was anything missing?*

2. What was it like to receive information about mental health in the health communication session?
 *Interviewer prompt: Please elaborate – what was good, what was missing, was it meaningful, why or why not?*

3. Would you prefer to receive information about mental health in another way? If so, how?

4. If a friend of yours had a child experiencing mental health problems, what advice would you give them?

5. How do you experience getting in contact with health care? Do you experience any challenges?

6. Is there any other kind of support that you think is important if your child were to struggle with mental health?
