# Supplementary material for: Bridging barriers: a mixed-methods study on using health communication to increase knowledge on mental health services among migrant parents in Sweden
Source: BMJ Public Health. 2026 Aug 5;4(3):e003272. doi: 10.1136/bmjph-2025-003272 (PMC13448519; doi:10.1136/bmjph-2025-003272)
Supplement: Supplementary file 6 [file bmjph-4-3-s006.docx]

# **Supplementary File 6. Coding tree interview data**

| Category | Subcategory | Example Quote |
| --- | --- | --- |
| Purpose comprehension | Limited understanding of activity objective | I did not fully understand the purpose of being there |
| Purpose comprehension | Understanding the objective of the activity | I learned how to deal with my children, grandchildren and how to talk to them |
| Knowledge growth and practical insights | Practical use of the knowledge gained from the activity | I now know where to turn to if I have problems |
| Knowledge growth and practical insights | New insights | I thought that the health center was for somatic illness only |
| Enhancing mental health literacy | Uncertainty of what mental health is | I knew a little about this mental distress but not that it was so widespread and that everyone could be affected |
| Enhancing mental health literacy | Religious strategies | Mental distress is taboo in Somalia, as a Muslim one has always believed it was the devil that causes mental symptoms to people who behaved badly |
| Enhancing mental health literacy | High mental health literacy | Seek Swedish health care which is good and understand much better rather than us doing 'Quran saar' |
| Enhancing mental health literacy | Not believing in the concept of mental health | I do not believe in mental distress, I do not want to be asked to participate again in the HC activity |
| Perceptions of and suggestions for improvement of the HC | Perceptions of the format | I wish there were more sessions |
| Perceptions of and suggestions for improvement of the HC | Perceptions of the format | I thought it was good to meet in a group and meet other parents and share thoughts with them |
| Perceptions of and suggestions for improvement of the HC | Perceptions of the content | What I missed was a person suffering from this [mental distress] that could explain to us how he/she felt |
| Perceptions of and suggestions for improvement of the HC | Suggestion of different target group | More appropriate for teenagers or mothers |
| Perceptions of health care access in Sweden | Overcoming language barriers | I have felt that health care staff helps us and there are physicians that speak Arabic |
| Perceptions of health care access in Sweden | Navigate in the Swedish health care system | I think it is difficult knowing when it is urgent or not, other than that I have not experienced challenges accessing health care |
| Perceptions of health care access in Sweden | Fear of not being listened to | In worst case they don't take care of you or they do not believe in you |

This table presents categories, subcategories, and example quotes from interview data, illustrating parent perceptions of the health communication intervention and access to mental health care.
